# Dental Amalgam Restorations and Children’s Neuropsychological Function: The New England Children’s Amalgam Trial

**DOI:** 10.1289/ehp.9497

**Published:** 2006-10-30

**Authors:** David C. Bellinger, David Daniel, Felicia Trachtenberg, Mary Tavares, Sonja McKinlay

**Affiliations:** 1 Department of Neurology, Children’s Hospital Boston and Harvard Medical School and; 2 Department of Environmental Health, Harvard School of Public Health, Boston, Massachusetts, USA; 3 Department of Psychology, University of Maine at Farmington, Farmington, Maine, USA; 4 New England Research Institutes, Watertown, Massachusetts, USA; 5 The Forsyth Institute, Boston, Massachusetts, USA

**Keywords:** children, dental amalgam, elemental mercury, neuropsychology, randomized controlled trial

## Abstract

**Background:**

A concern persists that children’s exposure to mercury vapor from dental amalgams produces neurotoxicity.

**Objective:**

Our goal was to compare the neuropsychological function of children, without prior exposure to dental amalgam, whose caries were repaired using either dental amalgam or mercury-free composite materials.

**Methods:**

We conducted a randomized controlled trial involving 534 6- to 10-year-old urban and rural children who were assessed yearly for 5 years using a battery of tests of intelligence, achievement, language, memory, learning, visual–spatial skills, verbal fluency, fine motor function, problem solving, attention, and executive function.

**Results:**

Although the mean urinary mercury concentration was greater among children in the amalgam group than the composite group (0.9 vs. 0.6 μg/g creatinine), few significant differences were found between the test scores of children in the two groups. The differences found were inconsistent in direction. Analyses using two cumulative exposure indices—surface years of amalgam and urinary mercury concentration—produced similar results.

**Conclusions:**

Exposure to elemental mercury in amalgam at the levels experienced by the children who participated in the trial did not result in significant effects on neuropsychological function within the 5-year follow-up period.

We previously reported on a randomized clinical trial, the New England Children’s Amalgam Trial (NECAT), in which no significant differences were found, over a 5-year follow-up interval, between the neuropsychological scores of children for whom dental amalgam was used to repair caries and the scores of children for whom mercury-free composite materials were used (Bellinger et al. 2006). The Full-Scale IQ score on the Wechsler Intelligence Scale for Children-Third Edition (WISC-III; Wechsler 1991) was the primary end point, and the General Memory Index (GMI) on the Wide Range Assessment of Memory and Learning (WRAML; Sheslow and Adams 1990), and the Visual Motor Composite (VMC) on the Wide Range Assessment of Visual–Motor Ability (WRAVMA; Adams and Sheslow 1995) were the two secondary end points. Each of these is a global score, derived by combining a child’s performance on tasks that assess somewhat different abilities.

The additional analyses reported in this article address three issues. First, if mercury vapor liberated from dental amalgams produces specific rather than general neuropsychological effects and are most likely to be evident on tests that assess specific cognitive domains, global scores such as Full-Scale IQ, GMI, and VMC might be relatively insensitive to important treatment-group differences. Even in the absence of treatment-group differences on global test scores, differences in specific cognitive domains could, depending on their nature and severity, represent morbidities with important consequences for children’s health and well-being. Therefore, here we report comparisons of the scores of the amalgam and composite groups on the sub-scales that contribute to Full-Scale IQ, the GMI, and the VMC, as well as scores on a battery of additional, domain-focused, neuropsychological and educational tests.

Second, the exposure index used in the primary analyses of the trial was treatment-group assignment. This could have introduced a form of exposure misclassification insofar as the variability in the treatment needs of the children in the amalgam group resulted in the receipt of variable amounts of amalgam and thus in their potential exposure to mercury. Therefore, we repeated the analyses replacing treatment-group assignment with two continuously distributed indices of exposure: surface-years of amalgam and urinary mercury concentration.

Third, it is possible that only a subset of children experienced adverse neuropsychological effects as the result of exposure to amalgam, either because of behaviors, such as bruxism or frequent gum chewing, that cause enhanced release of mercury (Barregard 2005; Barregard et al. 1995) or because of enhanced sensitivity to mercury. Two recent studies of dental professionals suggest that polymorphisms for brain-derived neurotrophic factor (Echeverria et al. 2005; Heyer et al. 2004) and the coproporphyrinogen oxidase gene (Echeverria et al. 2006) modify the neurotoxicity of elemental mercury. If the prevalence of such enhanced vulnerability to elemental mercury is low or the associated increase in neuropsychological toxicity is modest in magnitude, its impact on the mean scores of children in the amalgam group might not have been sufficiently large to produce significant treatment-group differences. In an attempt to identify the presence of a subgroup of children who are particularly sensitive to amalgam, we compared the distributional characteristics of the scores within the treatment groups.

## Methods

### Study design and participants

Children were eligible if they were 6–10 years of age, fluent in English, had no known prior or existing amalgam restorations, had two or more posterior teeth with dental caries, and did not have a physician-diagnosed psychological, behavioral, neurological, immunosuppressive, or renal disorder (Children’s Amalgam Trial Study Group 2003). Children were recruited from several community dental clinics in the Boston/Cambridge area of Massachusetts, an urban setting, and from a dental clinic in Farmington, Maine, a rural area.

A total of 5,116 children were screened for eligibility. Eligibility was confirmed for 598 children. At baseline visits, children received a dental examination by a study dentist, X rays, and standard preventive dental care (e.g., cleaning, application of sealants). Other baseline visit activities included the collection of blood and urine samples, anthropometric measurements of height, weight, and body fat, neuropsychological testing of the child, a health interview with the child’s guardian, and neuropsychological testing of the guardian.

After completion of baseline visits, children were randomized to a study treatment arm. Randomization was stratified by geographic location (Boston/Cambridge vs. Farmington) and number of teeth with caries (two to four vs. five or more), using randomly permuted blocks within each of the four strata.

The NECAT was conducted in accordance with all applicable requirements for the protection of human subjects. All children provided assent and parents provided informed consent. The study protocol was approved by the institutional review boards of the New England Research Institutes, the Forsyth Institute, and the clinics from which children were recruited.

### Interventions and follow-up

All children had semiannual dental examinations as well as additional visits required to meet treatment needs. For children in the amalgam arm, a dispersed-phase amalgam was used to restore all posterior teeth with caries at baseline and to repair incident caries during the 5-year trial period. For children in the composite arm, composite material (white filling) was used for all restorations. Following standard clinical practice, for both groups, composite material was used to repair caries in the front teeth, and stainless steel crowns were used to restore primary teeth with extensive lesions that could not otherwise be restored. The choices of dental materials and techniques were standardized across sites and dentists.

Urine samples were collected annually and analyzed for elemental mercury using cold vapor atomic absorption. Values were expressed as micrograms per gram creatinine. The analyses reported use only urinary mercury concentrations in samples collected at 3, 4, and 5 years of follow-up. After 1 February 2000, the detection limit, which had been 1.5 ng/mL, was reduced to 0.45 ng/mL as a result of increasing the volume of sample analyzed for each child. Samples with a mercury concentration below the detection limit were assigned a value of 0.45/√2 (Bellinger et al. 2006).

Participants and dentists could not be blinded to treatment assignment, but all individuals who collected outcome data (e.g., neuropsychological tests) or analyzed specimens (e.g., for mercury) were blinded to children’s treatment assignments.

### Neuropsychological assessments

At baseline, before randomization and the receipt of any dental treatments, children participated in two 3-hr neuropsychological test sessions. At the first session, the WISC-III (Wechsler 1991) and the Wechsler Individual Achievement Test (WIAT) (Psychological Corporation 1992) were administered. These tests were again administered at 3 and 5 years after baseline. The second baseline session consisted of a battery of domain-focused tests. This test battery, which was again administered at 1, 2, and 4 years after baseline, included the WRAML (Sheslow and Adams 1990), the WRAVMA (Adams and Sheslow 1995), the Trail-Making Test (Spreen and Strauss 1991), finger tapping (the WPS Electronic Tapping Test; Western Psychological Services, Los Angeles, CA), ordered and unordered verbal cancellation (Mesulam 1985), category fluency (McCarthy 1972), the Controlled Oral Word Association Test (letter fluency) (Spreen and Strauss 1991), simple visual reaction time (the Standard Reaction Timer; Software Science, Cincinnati, OH), the Stroop Color–Word Interference Test (Trenerry et al. 1989), and the Wisconsin Card Sorting Test (Heaton et al. 1993).

A total of 14 testers were used at the Boston/Cambridge site and five testers at the Farmington site. Quality control of the assessments was assured by having all examiners trained and certified by one supervising psychologist (D.C.B.) before conducting assessments of children enrolled in the trial, and monitored over the course of data collection. Each testing session was completely rescored by a second individual and errors were corrected. A variety of computerized algorithms were used to check the entered data for internal consistency.

### Sample size determination

The trial was designed to achieve 80% power to detect a 3-point difference between treatment arms in 5-year change in Full-Scale IQ score, adjusted for baseline IQ score and randomization stratum. Assuming a retention rate of 75% over the 5-year follow-up period, the recruitment goal was 250 children per treatment arm, for a total sample size of 500 children.

### Statistical analysis

We used intention-to-treat analyses, using analysis of covariance, to compare the amalgam and composite groups in terms of the changes, over 5 years, in scores on the WISC-III and WIAT and the changes, over 4 years, in scores on the domain-focused tests. These analyses thus indicated whether the central tendencies of the distributions of change scores differed in the amalgam and composite groups. Adjustments were made for baseline covariates: test score, randomization stratum, age, sex, socioeconomic status, hair mercury, and blood lead level. We calculated socioeconomic status using the method developed by Green (1970). Hair mercury was included to control for methylmercury, a form of mercury that is known to be a developmental neurotoxicant (Weiss 2006) but acquired primarily by consumption of contaminated seafood. Elevated blood lead level is a well-known developmental risk factor, with an increased prevalence among children who are socioeconomically disadvantaged (Bellinger 2006).

We evaluated children’s neuropsychological test scores in relation to two continuously distributed indices of exposure. The first index was surface-years of amalgam (number of amalgam surfaces weighted by number of years present), calculated from the information contained in dental clinic records regarding dates of amalgam placement, the number of tooth surfaces involved in the restoration, the timing of loss of primary teeth containing amalgam restorations, and the like. The second index was urinary mercury concentration. Scores on the WISC-III and WIAT, both of which were administered at year 5 of follow-up, were evaluated in relation to the mean of available urinary mercury concentrations at years 3, 4, and 5. Scores on the other tests, which were administered for the final time at year 4 of follow-up, were evaluated in relation to the mean of available urinary mercury concentrations at years 3 and 4 of follow-up. We evaluated the associations between these indices of exposure and children’s neuropsychological test scores using analysis of covariance, adjusting for the same set of baseline covariates used in the intention-to-treat analyses.

We conducted the analyses using the Kolmogorov-Smirnov test (Stuart and Ord 1991) to compare characteristics other than the central tendency of the distributions of the change scores in the treatment groups. When the results indicated that the change scores of children in the two groups did not come from the same distribution, we determined whether this was attributed to differences between treatment groups in the percentages of children with change scores indicating substantial deterioration of performance over the follow-up interval. Of particular interest was whether, in the absence of a treatment-group difference in mean change score, a greater percentage of children in the amalgam group than in the composite group demonstrated such deterioration.

To evaluate the impact of interexaminer variability on the results, we repeated the intention-to-treat analyses including adjustment for a set of indicator variables representing the neuropsychological examiners.

## Results

Table 1 shows that the treatment groups did not differ significantly in terms of age, race, household income, education of primary caregiver, Full-Scale IQ, hair and urinary mercury concentrations, blood lead level, and number of decayed tooth surfaces. Females outnumbered males in the composite arm. Children were primarily non-Hispanic white (62%), with non-Hispanic blacks making up an additional 19% of the sample. The mean number of total decayed tooth surfaces at baseline was 9.5, with 1.7 of the surfaces being in permanent teeth. Slightly more than half of the children (54%) had five or more teeth with caries that required restoration, with the rest having two to four carious teeth. Children from the Boston/Cambridge area tended to have more caries than did children from Farmington (10.3 vs. 8.6 carious surfaces, respectively).

Children continued to have dental treatment needs over the course of the 5-year follow-up period, averaging approximately one new filled surface per year. The treatment needs were similar in the treatment groups (Table 2). At the end of the follow-up period, the number of restored surfaces in place ranged from 0 to 36 in both treatment groups, and the mean values did not differ significantly (*p* = 0.16). The amalgam and composite groups also did not differ significantly in the cumulative number of restored surfaces over the trial (*p* = 0.10). In the amalgam group, 79% of the surfaces had been restored with amalgam. At the end of the 5-year follow-up period, the mean urinary mercury concentration was significantly greater among children in the amalgam group (0.9; range, 0.1–5.7 μg/g creatinine) than among children in the composite group (0.6; range, 0.1–2.9 μg/g creatinine) (*p* < 0.001).

Table 3 shows the change scores over the follow-up interval for each test score. Of all the change scores evaluated, only two differed significantly between the amalgam and composite treatment groups. On the Number–Letter Memory subtest of the WRAML, the 4-year change score of the amalgam group was significantly more positive than was the change score of the composite group, indicating greater improvement over time in the amalgam group. The 4-year change in the time required to complete Part B of the Trail-Making Test was significantly more negative in the composite group than in the amalgam group, indicating greater improvement over time in the composite group.

The results of analyses using surface-years of amalgam or urinary mercury concentration as the exposure metric were consistent with those of the intention-to-treat analyses, providing no evidence of a detrimental effect of amalgam on children’s test scores. The coefficient for surface-years of amalgam was significant for three scores (Picture Memory and Number–Letter Memory of the WRAML and letter fluency), but for all three scores, the sign was positive, indicating that the score improved with increasing exposure to amalgam (Table 4). Urinary mercury concentration was not significantly associated with any of the test scores (Table 5).

The results of the Kolmogorov-Smirnov tests indicated that the only two scores for which the distributions of changes scores in the amalgam and composite groups differed significantly were two subtests of the WRAML: Finger Windows and Number–Letter Memory (Table 3). On both tests, however, children in the amalgam group showed greater improvement over time than the children in the composite group, with the difference being significant for Number–Letter Memory. The distributions of change scores were not significantly different on the Trail-Making Test Part B (time to complete), the test on which the composite group showed significantly more improvement than the amalgam group.

Adjustment for neuropsychological examiner did not produce results that were appreciably different (data not shown).

## Discussion

These analyses revealed an absence of consistent differences between the scores of children in the amalgam and composite treatment groups on a battery of neuropsychological tests that assessed a wide range of domains, including intelligence, achievement, language, memory, learning, visual–spatial skills, verbal fluency, fine motor function, problem solving, attention, and executive function. The findings were similar when the dichotomous variable treatment-group assignment was replaced by two continuously distributed indices of exposure, one that combined the amount and duration of amalgam a child received and one that was a biomarker, urinary mercury concentration. Furthermore, no evidence was found to support the hypothesis that a subset of children in the amalgam group suffered substantial harm. The number of significant differences observed was similar to that which might have been expected to occur by chance.

Although neuropsychological deficits associated with amalgam exposure have been reported in several studies of dental professionals (Bittner et al. 1998; Echeverria et al. 1995, 1998; Ngim et al. 1992) and others exposed occupationally to mercury (Rohling and Demakis 2006), our findings are similar to those involving mercury exposure in cohorts drawn from the general population of adults and children (Brownawell et al. 2005). In a cross-sectional study of 550 30- to 49-year-old healthy employed adults, scores on tests of verbal memory, nonverbal memory, attention, psychomotor speed, and fine motor coordination were not significantly associated with any of several exposure indices considered (number of visible amalgam surfaces, number of visible occlusal amalgam surfaces, urinary mercury concentration) (Factor-Litvak et al. 2003). The mean urinary mercury concentration in that cohort of adults, 1.7 μg/g creatinine, was higher than the mean concentration of 0.9 μg/g creatinine among the children in the amalgam treatment group in our trial 5 years after placement of their first amalgam restorations. In a study of 1,663 Vietnam-era veterans, the total number of tooth surfaces with amalgam fillings was unrelated to clinical neurological signs (e.g., tremor, coordination, station, gait, strength, sensation, muscle stretch reflexes, or indices of peripheral neuropathy), although it was associated with vibrotacile sensation in non-diabetic participants (Kingman et al. 2005). In a study of 384 German 6-year-olds, 24-hr urinary excretion of mercury, which averaged 0.16 μg, was not significantly related to scores on a variety of tests, including the Vocabulary and Block Design subtests of the WISC and five tests of the computerized Neurobehavioral Evaluation System 2 (pattern comparison, pattern memory, tapping, simple reaction time, continuous performance test) (Walkowiak et al. 1998). In this cohort, some indices of visual contrast sensitivity did decline with increasing urinary mercury excretion, however (Altman et al. 1998).

Over the course of the follow-up interval, the scores of children in both treatment groups tended to change in the direction of improved performance, even on tests for which scores are standardized for age. Several factors might have contributed to improved performance over time. First, this could represent a type of sampling bias, reflecting the characteristics of families who are motivated to enroll in such a trial and to participate for its full duration. Second, all tests except the WISC-III and WIAT were administered yearly, so the general improvement in scores might reflect the familiarity that children developed with the test materials and expectations. Particularly large improvements tended to be on performance-based tests, such as the WRAVMA pegboard and the Processing Speed composite of the WISC-III, one component of which is Symbol Search, a timed task that involves matching symbols and digits. A substantial improvement was also noted on the WRAML Learning Index, which reflects the rapidity with which a child learns new material, such as sound–symbol pairs, a word list, and the locations of hidden designs. Repeated administration of these tasks, even at yearly intervals, might be expected to result in an increased rate of acquisition of the material.

As noted, the dental treatment needs of the children enrolled in the trial were substantial and exceeded those typical of the general population of U.S. children. For example, among 6- to 11-year-old children who participated in the National Health and Nutrition Examination Survey (NHANES) 1999–2002, the prevalence of dental caries in primary teeth was 22%, and the mean numbers of decayed or filled primary teeth and surfaces were 1.7 and 3.7, respectively (Beltran-Aguilar et al. 2005). The prevalence of dental caries in permanent teeth was 20% (mean number of decayed, missing, and filled teeth and surfaces in permanent teeth were 0.1 and 0.4, respectively) (Beltran-Aguilar et al. 2005). Therefore, children assigned to the amalgam group in the NECAT are likely to have experienced greater exposures to mercury vapor from amalgams than do most children in the United States. Given our failure to detect significant differences between the amalgam and composite groups in neuropsychological function, these results provide reassurance that the use of dental amalgam to repair caries is not producing substantial neuropsychological morbidity in the general population of children in the United States.

The conclusions must be tempered, first, by a recognition that the follow-up interval of 4–5 years might have been too short to allow for the expression of such deficits. Second, the critical window of children’s greatest vulnerability to elemental mercury might already have passed by the time the children were enrolled in the trial (≥ 6 years of age). Given the heightened sensitivity of the fetus to methylmercury, prenatal exposure to mercury vapor, which is known to cross the placenta, warrants increased attention. In the NHANES 1999–2000 survey, among women of child-bearing age, an increase of 10 dental surfaces restored with amalgam was associated with an estimated increase of 1.8 μg/L in urinary mercury concentration (Dye et al. 2005). Mercury level in amniotic fluid is weakly associated with number of amalgam fillings (Luglie et al. 2005). A recent case–control study did not, however, find an increased risk of delivering a low-birth-weight infant among women who had up to 11 amalgam restorations placed during pregnancy (Hujoel et al. 2005). The results of studies of the reproductive outcomes of women with dental workplace exposures have been mixed (Dahl et al. 1999; Elghany et al. 1997; Ericson and Kallen 1989). In some of these studies, distinguishing the potential impact of mercury exposure from the impacts of other workplace exposures, such as to disinfectants containing ethanol and benzene, is difficult. Third, the prevalence of children with enhanced sensitivity to elemental mercury might be too low among the children enrolled in the NECAT for us to have been able to detect their effects on the distribution of responses. Fourth, children with preexisting neuropsychological or behavioral disorders were not eligible for enrollment. Our findings therefore do not provide any information about the possibility that amalgam-related exposure to mercury vapor might exacerbate such disorders. Nevertheless, our results indicate that even among children with substantial dental needs, an increased risk of neuropsychological deficits could not be detected among children whose dental restorations contained elemental mercury.

## Figures and Tables

**Table 1:** Baseline characteristics of trial participants, by treatment group.

Characteristic	Amalgam (*n* = 267)	Composite (*n* = 267)
Site [no. (%)]		
Boston/Cambridge	144 (53.9)	147 (55.1)
Farmington	123 (46.1)	120 (44.9)
No. of carious surfaces [mean ± SD (range)]	9.8 ± 6.9 (2–39)	9.3 ± 6.2 (2–36)
Age (years) [mean ± SD (range)]	7.9 ± 1.3 (5.9–11.4)	7.9 ± 1.4 (5.9–11.5)
Sex [no. (%)]		
Female	131 (49.1)	156 (58.4)
Male	136 (50.9)	111 (41.6)
Race [no. (%)]^a^		
Non-Hispanic white	165 (64.0)	158 (60.3)
Non-Hispanic black	49 (19.0)	49 (18.7)
Hispanic	15 (5.8)	23 (8.8)
Other	29 (11.2)	32 (12.2)
Household income [no. (%)]		
≤ $20,000	74 (29.2)	86 (33.1)
$20,000–$40,000	113 (44.7)	109 (41.9)
> $40,000	66 (26.1)	65 (25.0)
Education of primary caretaker [no. (%)]		
< High school	34 (13.2)	38 (14.6)
High school graduate	197 (76.4)	194 (74.3)
College graduate	18 (7.9)	17 (6.5)
Postgraduate degree	9 (3.5)	12 (4.6)
WISC-III Full-Scale IQ [mean ± SD (range)]	95.1 ± 14.5 (65–141)	96.1 ± 12.1 (62–123)
Urinary mercury ≥ 1.5 ng/mL [no. (%)]	21 (8.4)	11 (4.5)
Hair mercury (μg/g) [mean ± SD (range)]	0.4 ± 0.5 (0.1–4.4)	0.4 ± 0.5 (0.1–4.5)
Blood lead (μg/dL) [mean ± SD (range)]	2.4 ± 1.9 (1–13)	2.3 ± 1.5 (1–11)

a Race was self-reported by parents.

**Table 2:** Dental treatment and amalgam exposure at the end of the 5-year follow-up period, by treatment group [mean ± SD (range)].

Dental treatment	Amalgam	Composite
No. of restored surfaces in mouth	5.3 ± 5.2 (0–36)*	6.1 ± 6.0 (0–36)*
No. of restored amalgam surfaces^a^	4.0 ± 4.0 (0–21)	0.05 ± 0.6 (0–9)
Cumulative no. of surfaces restored (over 5 years)^b^	14.8 ± 9.5 (2–55)**	16.0 ± 9.8 (2–51)**
Cumulative no. of surfaces restored with amalgam^b^	11.7 ± 7.0 (0–35)	0.05 ± 0.6 (0–9)

a Two children in the composite group received amalgam restorations from an out-of-study dentist.

b Cumulative numbers do not include children who withdrew from the study.

* *p* = 0.16 for difference between amalgam and composite groups.

** *p* = 0.10 for the difference between amalgam and composite groups.

**Table 3:** Changes in test scores between baseline and 4- or 5-year follow-up, by treatment group [adjusted coefficient ± SE (*n*)].

			*p*-Value	
Test score	Amalgam	Composite	Intention-to-treat^a^	Kolmogorov-Smirnov Test
WISC-III				
Factor				
Verbal Comprehension	2.2 ± 0.6 (219)	1.5 ± 0.6 (217)	0.46	0.69
Perceptual Organization	3.6 ± 0.7 (219)	3.1 ± 0.7 (216)	0.58	0.72
Freedom from Distractibility	3.9 ± 0.7 (219)	2.4 ± 0.7 (216)	0.57	0.71
Processing Speed	7.2 ± 0.9 (216)	5.1 ± 0.9 (217)	0.08	0.09
Subtest				
Verbal				
Information	0.3 ± 0.2 (219)	0.4 ± 0.2 (217)	0.61	0.97
Similarities	0.8 ± 0.1 (219)	0.7 ± 0.2 (217)	0.56	1.00
Vocabulary	0.4 ± 0.1 (219)	0.2 ± 0.1 (217)	0.26	0.41
Comprehension	0.2 ± 0.2 (219)	−0.1 ± 0.2 (217)	0.22	0.98
Digit Span	0.7 ± 0.2 (219)	0.5 ± 0.2 (216)	0.26	1.00
Performance				
Picture Completion	1.2 ± 0.2 (219)	1.2 ± 0.2 (217)	0.84	0.57
Coding	0.2 ± 0.2 (218)	−0.1 ± 0.2 (217)	0.19	0.81
Picture Arrangement	0.5 ± 0.2 (219)	0.6 ± 0.2 (216)	0.79	0.77
Block Design	0.3 ± 0.2 (219)	0.1 ± 0.2 (217)	0.43	0.63
Object Assembly	0.3 ± 0.2 (219)	0.3 ± 0.2 (216)	0.96	0.99
Symbol Search	2.5 ± 0.2 (216)	2.2 ± 0.2 (217)	0.21	0.73
Mazes	0.8 ± 0.2 (218)	0.7 ± 0.2 (217)	0.85	0.79
WIAT				
Composite				
Reading	−1.0 ± 0.7 (217)	−1.7 ± 0.7 (215)	0.44	0.34
Mathematics	−1.9 ± 0.7 (216)	−3.0 ± 0.8 (207)	0.33	0.57
Scale				
Basic Reading	−0.6 ± 0.6 (219)	−1.3 ± 0.6 (216)	0.37	0.53
Reading Comprehension	0.7 ± 0.7 (217)	0.2 ± 0.7 (215)	0.70	0.26
Numerical Operations	−5.2 ± 0.8 (216)	−6.7 ± 0.9 (207)	0.20	0.72
Math Reasoning	1.5 ± 0.7 (219)	1.3 ± 0.7 (216)	0.85	1.00
Spelling	−1.7 ± 0.7 (219)	−3.1 ± 0.7 (215)	0.14	0.81
Listening Comprehension	−5.5 ± 0.7 (212)	−4.5 ± 0.7 (205)	0.27	0.63
WRAML				
Index				
Verbal Memory	2.9 ± 0.6 (212)	2.2 ± 0.6 (202)	0.47	0.57
Visual Memory	6.3 ± 0.8 (212)	5.0 ± 0.8 (204)	0.23	0.42
Learning	10.2 ± 0.8 (212)	10.3 ± 0.8 (203)	0.91	0.28
Scale				
Picture Memory	0.8 ± 2.2 (212)	0.6 ± 0.2 (204)	0.28	0.98
Design Memory	1.0 ± 0.2 (212)	1.2 ± 0.2 (204)	0.53	0.95
Story Memory	0.7 ± 0.2 (212)	0.9 ± 0.2 (203)	0.46	0.96
Verbal learning	1.4 ± 0.2 (212)	1.4 ± 0.2 (203)	0.92	0.84
Finger Windows	0.8 ± 0.2 (212)	0.4 ± 0.2 (204)	0.09	0.05
Sound Symbol	2.8 ± 0.2 (212)	2.7 ± 0.2 (203)	0.55	0.12
Sentence Memory	0.3 ± 0.1 (212)	0.3 ± 0.1 (204)	0.80	0.56
Visual Learning	0.4 ± 0.2 (212)	0.6 ± 0.2 (203)	0.29	0.82
Number–Letter Memory	0.3 ± 0.1 (212)	−0.3 ± 0.1 (203)	0.002	0.04
WRAVMA				
Drawing	−3.8 ± 0.9 (211)	−3.1 ± 0.9 (203)	0.63	0.78
Matching	3.0 ± 0.8 (211)	3.5 ± 0.8 (203)	0.62	0.96
Pegboard	9.3 ± 0.9 (211)	8.4 ± 1.0 (203)	0.50	0.82
Trail-Making Test				
Part A: time to complete	−15.5 ± 0.3 (203)	−16.1 ± 0.3 (200)	0.25	0.68
Part A: no. of errors	−0.2 ± 0.03 (203)	−0.1 ± 0.04 (200)	0.07	0.95
Part B: time to complete	−45.6 ± 1.0 (201)	−50.4 ± 1.1 (193)	0.002	0.36
Part B: no. of errors	−0.6 ± 0.1 (201)	−0.7 ± 0.1 (193)	0.09	0.88
Finger Tapping				
Right hand (mean of 5 trials)	5.7 ± 0.4 (208)	5.1 ± 0.4 (202)	0.28	0.42
Left hand (mean of 5 trials)	6.4 ± 0.3 (208)	5.7 ± 0.3 (202)	0.10	0.54
Verbal Cancellation				
Ordered trial: no. of errors	−19.3 ± 0.2 (200)	−19.4 ± 0.2 (198)	0.57	0.59
Unordered trial: no. of errors	−15.0 ± 0.1 (200)	−14.9 ± 0.2 (198)	0.83	0.99
Verbal Fluency				
Category fluency (sum of 4 trials)	9.7 ± 0.4 (210)	9.5 ± 0.4 (201)	0.70	0.91
Letter fluency (sum of 3 trials)	13.3 ± 0.5 (210)	12.4 ± 0.6 (201)	0.25	0.77
Reaction Time				
Mean response time (msec)	−0.1 ± 0.0 (181)	−0.1 ± 0.0 (180)	0.67	0.79
Response time SD	−0.1 ± 0.0 (181)	−0.1 ± 0.0 (180)	0.74	0.91
Stroop Color–Word Interference Test				
Color	19.7 ± 0.7 (165)	18.0 ± 0.7 (164)	0.09	0.17
Word	26.1 ± 0.8 (165)	25.0 ± 0.8 (163)	0.35	0.89
Color–Word	12.8 ± 0.6 (165)	12.8 ± 0.6 (164)	0.96	0.75
Wisconsin Card Sorting Test				
No. of categories achieved	1.1 ± 0.1 (200)	1.1 ± 0.1 (194)	0.94	0.97
No. of trials to first category	−5.8 ± 0.3 (200)	−5.6 ± 0.3 (194)	0.69	0.96
Total errors^b^	15.6 ± 0.9 (199)	17.2 ± 1.0 (191)	0.25	0.14
Total perseverative errors^b^	17.6 ± 0.9 (199)	19.3 ± 1.0 (191)	0.19	0.59
Percent conceptual level responses	15.7 ± 1.0 (199)	17.3 ± 1.0 (191)	0.26	0.20

a Adjusted for baseline score, randomization stratum, baseline age, sex, baseline family socioeconomic status, baseline hair mercury concentration, and baseline blood lead concentration.

b Standard score.

**Table 4:** Associations between neuropsychological test scores and surface-years of exposure to amalgam restorations [adjusted coefficient ± SE (*n*)].

Test score	Surface-years of amalgam	*p*-Value^a^
WISC-III		
Factor		
Verbal Comprehension	0.01 ± 0.02 (434)^b^	0.48
Perceptual Organization	0.00 ± 0.02 (433)	0.94
Freedom from Distractibility	0.02 ± 0.02 (433)	0.50
Processing Speed	0.03 ± 0.03 (431)	0.28
Subtest		
Verbal		
Information	−0.00 ± 0.01 (434)	0.74
Similarities	0.00 ± 0.01 (434)	0.83
Vocabulary	0.01 ± 0.00 (434)	0.19
Comprehension	0.01 ± 0.01 (434)	0.33
Digit Span	0.01 ± 0.01 (433)	0.26
Performance		
Picture Completion	0.01 ± 0.01 (434)	0.41
Coding	0.00 ± 0.01 (433)	0.47
Picture Arrangement	−0.00 ± 0.01 (433)	0.46
Block Design	0.00 ± 0.01 (434)	0.58
Object Assembly	−0.00 ± 0.01 (433)	0.66
Symbol Search	0.01 ± 0.01 (431)	0.29
Mazes	0.01 ± 0.01 (433)	0.35
WIAT		
Composite		
Reading	0.03 ± 0.02 (430)	0.20
Mathematics	0.03 ± 0.03 (421)	0.33
Scale		
Basic Reading	0.03 ± 0.02 (433)	0.16
Reading Comprehension	0.02 ± 0.02 (430)	0.41
Numerical Operations	0.03 ± 0.03 (421)	0.29
Math Reasoning	0.01 ± 0.02 (433)	0.75
Spelling	0.03 ± 0.02 (432)	0.21
Listening Comprehension	−0.03 ± 0.02 (415)	0.19
WRAML		
Index		
Verbal Memory	0.01 ± 0.03 (406)	0.73
Visual Memory	0.05 ± 0.03 (408)	0.10
Learning	0.02 ± 0.03 (407)	0.64
Scale		
Picture Memory	0.02 ± 0.01 (408)	0.008
Design Memory	−0.01 ± 0.01 (408)	0.48
Story Memory	−0.00 ± 0.01 (407)	0.57
Verbal Learning	0.00 ± 0.01 (407)	0.99
Finger Windows	0.01 ± 0.01 (408)	0.14
Sound Symbol	0.01 ± 0.01 (407)	0.26
Sentence Memory	−0.00 ± 0.01 (408)	0.52
Visual Learning	−0.00 ± 0.01 (407)	0.67
Number–Letter Memory	0.01 ± 0.01 (407)	0.01
WRAVMA		
Drawing	−0.02 ± 0.04 (406)	0.56
Matching	−0.04 ± 0.03 (406)	0.27
Pegboard	0.03 ± 0.04 (406)	0.45
Trail-Making Test		
Part A: time to complete	0.02 ± 0.01 (395)	0.22
Part A: no. of errors	−0.00 ± 0.00 (395)	0.26
Part B: time to complete	0.06 ± 0.04 (387)	0.15
Part B: no. of errors	0.00 ± 0.00 (387)	0.38
Finger Tapping		
Right hand (mean of 5 trials)	0.01 ± 0.01 (402)	0.53
Left hand (mean of 5 trials)	0.01 ± 0.01 (402)	0.42
Verbal Cancellation		
Ordered trial: no. of errors	0.00 ± 0.01 (367)	0.88
Unordered trial: no. of errors	0.00 ± 0.01 (367)	0.77
Verbal Fluency		
Category fluency (sum of 4 trials)	0.00 ± 0.02 (404)	0.84
Letter fluency (sum of 3 trials)	0.04 ± 0.02 (404)	0.05
Reaction Time		
Mean response time (msec)	−0.00 ± 0.00 (356)	0.62
Response time SD	0.00 ± 0.00 (356)	0.46
Stroop Color–Word Interference Test		
Color	0.01 ± 0.03 (323)	0.70
Word	0.00 ± 0.03 (322)	0.97
Color–Word	−0.00 ± 0.02 (323)	0.87
Wisconsin Card Sorting Test		
No. of categories achieved	−0.00 ± 0.00 (387)	0.50
No. of trials to first category	−0.00 ± 0.01 (387)	0.92
Total errors^c^	−0.04 ± 0.04 (383)	0.24
Total perseverative errors^c^	−0.05 ± 0.04 (383)	0.20
Percent conceptual level responses	−0.04 ± 0.04 (383)	0.29

a Adjusted for baseline score, randomization stratum, baseline age, sex, baseline family socioeconomic status, baseline hair mercury concentration, and baseline blood lead concentration.

b Coefficient represents the change in test score for each unit increase in surface-years of amalgam.

c Standard score.

**Table 5:** Associations between neuropsychological test scores and urinary mercury concentration [adjusted coefficient ± SE (*n*)].

Test score	Urinary mercury	*p*-Value^a^
WISC-III		
Factor		
Verbal Comprehension	0.40 ± 0.58 (433)^b^	0.50
Perceptual Organization	0.65 ± 0.64 (432)	0.31
Freedom from Distractibility	−0.37 ± 0.69 (432)	0.60
Processing Speed	0.01 ± 0.80 (430)	0.99
Subtest		
Verbal		
Information	0.02 ± 0.16 (433)	0.89
Similarities	−0.02 ± 0.13 (433)	0.91
Vocabulary	0.17 ± 0.13 (433)	0.19
Comprehension	−0.00 ± 0.18 (433)	0.99
Digit Span	0.04 ± 0.15 (432)	0.80
Performance		
Picture Completion	0.01 ± 0.18 (433)	0.94
Coding	−0.01 ± 0.19 (432)	0.97
Picture Arrangement	−0.01 ± 0.18 (432)	0.97
Block Design	0.07 ± 0.16 (433)	0.66
Object Assembly	0.29 ± 0.17 (432)	0.08
Symbol Search	0.04 ± 0.19 (430)	0.83
Mazes	−0.11 ± 0.19 (432)	0.55
WIAT		
Composite		
Reading	0.17 ± 0.63 (429)	0.79
Mathematics	0.57 ± 0.70 (420)	0.42
Scale		
Basic Reading	0.23 ± 0.58 (432)	0.69
Reading Comprehension	0.32 ± 0.66 (429)	0.63
Numerical Operations	0.20 ± 0.78 (420)	0.79
Math Reasoning	0.58 ± 0.67 (432)	0.39
Spelling	0.38 ± 0.63 (431)	0.54
Listening Comprehension	−0.52 ± 0.62 (414)	0.40
WRAML		
Index		
Verbal Memory	0.31 ± 0.53 (400)	0.56
Visual Memory	0.79 ± 0.65 (402)	0.23
Learning	0.57 ± 0.68 (401)	0.40
Scale		
Picture Memory	0.08 ± 0.13 (402)	0.52
Design Memory	0.01 ± 0.16 (402)	0.96
Story Memory	0.10 ± 0.15 (401)	0.52
Verbal Learning	0.11 ± 0.15 (401)	0.49
Finger Windows	0.18 ± 0.14 (402)	0.21
Sound Symbol	0.05 ± 0.15 (401)	0.74
Sentence Memory	0.04 ± 0.12 (402)	0.77
Visual Learning	0.06 ± 0.15 (401)	0.67
Number–Letter Memory	−0.03 ± 0.12 (401)	0.82
WRAVMA		
Drawing	0.33 ± 0.76 (400)	0.66
Matching	−0.25 ± 0.68 (400)	0.71
Pegboard	0.23 ± 0.79 (400)	0.77
Trail-Making Test		
Part A: time to complete	0.07 ± 0.30 (390)	0.83
Part A: no. of errors	−0.03 ± 0.03 (390)	0.37
Part B: time to complete	−0.26 ± 0.94 (382)	0.78
Part B: no. of errors	0.03 ± 0.05 (382)	0.51
Finger Tapping		
Right hand (mean of 5 trials)	−0.42 ± 0.31 (396)	0.17
Left hand (mean of 5 trials)	−0.22 ± 0.27 (396)	0.41
Verbal Cancellation		
Ordered trial: no. of errors	0.01 ± 0.19 (387)	0.98
Unordered trial: no. of errors	−0.08 ± 0.13 (387)	0.52
Verbal Fluency		
Category fluency (sum of 4 trials)	−0.17 ± 0.36 (398)	0.63
Letter fluency (sum of 3 trials)	0.12 ± 0.45 (398)	0.80
Reaction Time		
Mean response time (msec)	0.00 ± 0.00 (352)	0.27
Response time SD	−0.00 ± 0.01 (352)	0.91
Stroop Color–Word Interference Test		
Color	−0.90 ± 0.62 (318)	0.14
Word	−0.82 ± 0.72 (317)	0.26
Color–Word	−0.04 ± 0.50 (318)	0.94
Wisconsin Card Sorting Test		
No. of categories achieved	−0.07 ± 0.05 (383)	0.15
No. of trials to first category	−0.02 ± 0.26 (383)	0.93
Total errors^c^	−0.63 ± 0.77 (379)	0.41
Total perseverative errors^c^	−0.16 ± 0.76 (379)	0.84
Percent conceptual level responses	−0.45 ± 0.81 (379)	0.58

a Adjusted for baseline score, randomization stratum, baseline age, sex, baseline family socioeconomic status, baseline hair mercury concentration, and baseline blood lead concentration.

b Coefficient represents the change in test score for each unit increase in urinary mercury concentration.

c Standard score.
